# An Account of the Late Captain M******'s Case

**Published:** 1783

**Authors:** 


### SECTION II.

### Essays and Observations.

I. *An Account of the late Captain M******'s Caſe. Communicated to Dr.* Simmons, F.R.S. *by Mr.* Charles White, F.R.S. *Surgeon to the Infirmary, and Lunatic Hoſpital, at Mancheſter.*

ON Monday the 10th of March 1783, about nine o'clock in the evening, Captain M—, aged 23, received a wound on the anterior part of the right axilla, juſt below the inferior edge of the pectoral muſcle, where it grows tendinous, by a cut and thruſt ſword, which paſſed under the the ſhoulder joint, and came out betwixt the neck of the humerus, and the neck of the ſcapula at its coſta inferior. The ſword was about an inch broad, and had paſſed tranſverſely, reſpecting the line of direction of the axillary artery and nerve; the thruſt was therefore moſt probably given in *quarte*. A prodigious effuſion of blood enſued. He was put into a chair, and ſoon fainted away, to ſuch a degree, that he appeared to many perſons, both of the faculty and others, to be really dead. But being obſerved to breathe, he was laid down upon a feather bed, and the wound examined. The bleeding was much checked by the fainting, which probably ſaved his life for the preſent.

The wound was enlarged both upwards and downwards, and the blood was diſcovered to come from an artery, and from its ſize, ſituation and direction, no doubt could be entertained of its being the axillary. An attempt was made to take up the artery, by paſſing a crooked needle under it; but this not ſucceeding, a needle was paſſed, on each ſide of the divided artery, through the integuments, bringing both ends of the ligature through the ſkin, at ſome diſtance above the wound, incloſing a good deal of ſubſtance. This ligature, being tied, tied, effectually fecured the veffel, in about half an hour after the accident happened. During the time this operation was performing, a ftrong compreffion was made above the clavicle, upon the artery, to prevent a further effufion of blood.

After the hæmorrhage was ftopped, fpirit of hartfhorn and water were given him, and he began to recover very gradually from his fainting; but though his pulfe returned in the found arm, which was perfectly warm, yet the injured arm continued cold and ufelefs, and not the leaft pulfation could be perceived in it. This unfortunate gentleman had a tender conftitution and a weak frame, much impaired by living in a hot climate, and by intemperance. He was fo intoxicated at the time of the accident, as to be now deprived of all fenfe, and fo exhaufted with the lofs of blood, that the fmall remains of life feemed to hang by a very flender thread. Under thefe circumftances, amputation was fo much out of the queftion, as never to have been once hinted at that evening, though there were half a dozen of the faculty prefent.—He paffed a reftlefs night, with ficknefs and puking, notwithftanding he took frequently of a neutral julep along with an opiate. He had likewife frequent fwoonings, and once entirely fainted away.

Tuefday morning, a fmall hæmorrhage returned, upon altering his pofition, which by its colour appeared to be from an artery: it ftopped by preffure upon the artery, above the clavicle, and the ligature, which was become fomewhat flacker, was made tighter by a bolfter being put under it. He was very thirfty, and drank large quantities of toaft and water, and other fmall liquids, which conftantly came up again, and it was with great difficulty he could be reftrained from drinking, as he had always been accuftomed to treat himfelf in that manner after intoxication. There was neither voluntary motion, nor fenfation, below the elbow. The thumb and fore-finger were flaccid, the other three fingers bent and rigid. The upper part of the arm was warm, but below the elbow perfectly cold, and no pulfation in the wrift. His pukings were very conftant. He took neutral draughts in the act of effervefcence, joined with an opiate. Another gentleman, being called into confultation, faid, before he faw the patient, that if the axillary artery was cut he fhould advife immediate amputation; but, upon feeing the weak ftate of of the patient, he gave it up, as totally unadvifable, nor was it ever afterwards propofed.

Tuefday evening, pulfe in the found arm 92 in a minute: fome of the faculty thought they could perceive a pulfation in the wounded arm, but in this they were not agreed. The hand and lower arm remained cold. Wine and brandy were given him frequently, in fmall quantities. The vomiting continued fo conftant, that no nutriment had ftayed upon his ftomach. He took feveral dofes of the tinctures of columbo and bark. His ftomach, belly, and arm were fomented with brandy.

Wednefday morning, he had had a bad night, notwithftanding the ufe of opiates; the lower arm had acquired fome degree of warmth, but no pulfation could be perceived in it, nor was there any motion or fenfation. The whole arm being a little fwelled, the ligature was flackened by taking away the bolfter. He took oil of cinnamon and columbo in draughts.—Wednefday noon, he was rather better.——Wednefday evening, the arm was more fwelled, and he appeared much worfe. Pulfe 140. As fo much fubftance was included within the ligature, it was determined, at a confultation, to untie it, in order to remove every thing that could occafion tenſion or irritation; and in hopes, as near forty-eight hours were expired, that the extremity of the artery was ſo far cloſed, as to be able to reſiſt the impetus of the blood, in the then languid ſtate of the circulation *. But the ligature was not taken out; it was cloſely and conſtantly watched, for a conſiderable time, by the ſurgeons or their pupils, but neither hæmorrhage, pulſation, ſenſation, nor motion below the wriſt, enſued. What little motion he had above, ſeemed to proceed from the deltoid muſcle. The ſtomach ſtill rejected every kind of nutriment.

Thurſday morning, the right arm was perfectly warm to the fingers ends, but no pulſation could be perceived. The veins, particularly the external radial, which is a continuation of the cephalic, were full and turgid with blood; when emptied, by ſtroaking them, they immediately filled again, from the extremity of the limb. He ſtill continued to puke up every kind of food. He took muſk and ſalt of hartſhorn,

* Arteries, by their own contractile power, eſpecially when aſſiſted by ligature, generally cloſe much ſooner than is expected; and if a tumefaction of the ſurrounding parts follow, it is a further ſecurity to them. But when they ſtop by coagulum, if that coagulum be forced away, the hæmorrhage will return.

which ftayed upon his ftomach. The limb was directed to be frequently rubbed.——Thurfday afternoon, a little falep in fage-tea and brandy ftayed with him, and he feemed rather better, and flept very comfortably; but there was a bloody ichorous difcharge from the wound.— Thurfday evening, he was much worfe: pulfe 152. A ftool was procured by a broth glyfter. He was very weak, and complained of his fhoulder, which was fomented with common fomentation and fpt. vin. camph.

Friday morning, he was very ill, but remained fenfible, and complained of difficulty of breathing. His vomiting returned. The arm was very warm, but had no perceptible pulfation. A flight, dufky inflammation, about the fize of a crown piece, appeared on the back part of the arm, below the wound. The pulfe in the left arm was fo weak and quick, as not to be counted. He took julepum vitæ with apparent fatisfaction.—Friday evening, about half paft feven, he expired.

*Diffection.*——On Saturday morning his body being examined, the fhoulder was found very livid and putrid. The thorax was opened, and the fubclavian artery injected; when the injection immediately ran out of the wound in the axilla.

axilla. Upon diſſecting the parts, it was found, that the axillary artery was totally divided below the ramus arteriæ circumflexæ humeri anterior. One of the ſix brachial nerves was alſo totally divided, and another ſo nearly cut through, as to hang by a very ſmall fibre. The ligature, which was left ſlack in the parts, was found to have ſecured the end of the divided artery, and included three of the nerves. The vein, which accompanies them, was wounded, but not included in the ligature.

*Remarks.*——If it had not been for the extreme loſs of blood, and the debilitated and irritable ſtate of the ſtomach, it is not impoſſible, that not only this gentleman's life might have been ſaved, but even the limb preſerved, and have been of ſome little uſe to him. It has frequently happened, when patients have been too much exhauſted by very great and ſudden effuſions of blood, that they have died in conſequence of the hæmorrhage, four or five days after the blood had been ſtopped. It was curious to obſerve the progreſſive and gradual approach of warmth into the limb, which was perfect, and continued to the laſt. The warmth of the limb was ſucceeded by a fulneſs and turgency of the veins, which, when ſtroaked to empty them of the the blood, immediately filled again. Thefe were evident proofs, that the limb was fufficiently fupplied with blood, which had found a new courfe; and want of circulation could not therefore be the occafion of his death.

Senfation dawned upon him in the fame gradual and progreffive manner, till it had got to the wrift, but no further. The warmth was all along prior to fenfation, fo that the blood found a paffage fooner than the nerves could exert their influence. Amputation could not therefore have faved his life, but moft probably would have haftened his death. The brachial artery has been tied above its divifion into the radial, ulnary, and interoffeal, and almoft three inches of it deftroyed, and yet the ufe of the limb was preferved *. The femoral artery has been tied in the thigh, in the Manchefter Infirmary, by Dr. Burchall †; and, in another cafe, above the middle of the thigh by Mr. Charles Leflie, of Cork ‡; both of which operations were attended with the fame happy confequences.

* See White's Cafes in Surgery, p. 139, where the means by which this is brought about are explained.

† Med. Obf. and Inq. vol. III. p. 106.

‡ Med. Com. vol. II. p. 176.

Mr. Warner || has given us the following cafe: "C. D. was afflicted with a caries of the joint "of the elbow, which was attended with fuch "circumftances as rendered the amputation of "the limb neceffary. The operation was per-"formed at a proper diftance above the difeafed "part, and the veffels were taken up by the "needle and ligatures.

"In a few days after the operation, the bra-"chial artery became fo dilated above the liga-"ture, as to endanger its burfting. Upon this "account it was judged neceffary to perform "the operation for the aneurifm, which was "done, and the veffel was fecured by ligature "above the upper extremity of its diftended "coats. After this operation every thing went "feemingly well on for fome time, when fud-"denly the artery appeared again dilated, and "was in danger of burfting above the fecond "ligature: thefe circumftances made it neceffary "to repeat the operation for the aneurifm. "From this time every thing went on fuccefs-"fully, till the ftump was upon the point of "being healed; when, quite unexpectedly, the "artery appeared a third time difeafed in the "fame manner as before; for which reafon a "third operation for the aneurifm was deter-"mined upon, and performed. The laft opera-"tion was near to the axilla; the patient con-"tinued well from this time, without any "relapfe."

|| Cafes in Surg. p. 128.

He

He further ſays, " The nouriſhment of the

" ſtump may be accounted for from the ramifi-

" cations ariſing from the principal trunk about

" the axilla, which becoming dilated, in pro-

" portion to the reſiſtance the blood meets with

" in its paſſage through the brachial artery, were

" found ſufficiently numerous and large enough

" to convey a proper ſupply to the parts be-

" neath."

About ſixty years ago, Mr. Hall was called to a man in Cheſhire, who had received a very conſiderable wound, juſt below the axilla, by a ſcythe, which had divided the brachial artery. The man ſoon fainted away with the loſs of blood, which preſerved his life, as nobody was near him. Mr. Hall being only accidentally in the neighbourhood, had no needles with him; but as ſoon as he arrived, he eaſily took hold of the artery with his finger and thumb, till he could procure ſome thread, which he immediately tied round the veſſel, and effectually ſecured it. The man recovered the uſe of his arm, though he had ever after a weak trembling pulſe in that arm.

Boerhaave mentions a very remarkable caſe of a peaſant, near Leyden, who had an axillary artery totally divided with a knife, upon which accident a great effuſion of blood enſued, and the patient fainted. The mouth of the veſſel retracted ſo far, that it was impoſſible to come at it with a ligature, or ſtop the hæmorrhage by any other means: and in this ſad and hopeleſs condition the poor man was abandoned to his fate. He continued ſeveral days in a languid

 ſtate, ftate, apparently ready to expire every moment; in which time nature performed what the furgeons could not, by clofing up the mouth of the divided artery. The arm decayed and gradually fhrunk and dried, becoming at length a rigid piece of mummy, which he carried about a long while.

Heifter * relates the cafe of a foldier who had the brachial artery wounded, a hand's breadth above the bend of the elbow, which was taken up by ligature, a finger's breadth above the puncture, and the patient recovered.

Ætius † recommends the tying of the brachial artery, three or four fingers below the armpit, and afterwards the dividing of it, by way of preparation for the operation of the aneurifm. The ufe of the tourniquet has now made this cruel operation totally unneceffary. In a number of cafes, where the brachial artery has been tied with fuccefs, the pulfation in the wrift has returned, in fome in half an hour, and, in others, on the firft, fecond, third, or even the feventeenth day; and, though in fome it has never returned, the patients recovered and had the perfect ufe of their arms.

In true aneurifms, and thofe of the mixed kind, by much the greater number cannot be cured without amputation, as they are frequently attended with a caries of the bone and deftruction of the neighbouring foft parts, and the artery itfelf is difeafed to a confiderable height. But, in the falfe aneurifm, whether the accident

* Ob. XLVIII. p. 54. † P. 810.

be be a recent one, or have been of fome ftanding, or whether there have been a puncture of the artery, or a total divifion of it, or wherever fituated, if the artery has been fairly and properly taken up by ligature, amputation has very rarely proved neceffary, and will feldom be found fo, if the patient be not too far advanced in years. Was it not therefore poffible, in Capt. M——'s cafe, that if he had had no other difficulties to ftruggle with, fuccefs might have attended the tying of the principal artery, as high as the axilla? for there are no confiderable branches betwixt the humerales circumflexæ, and the divifion near the elbow; and in this cafe the artery was divided below thofe branches. The fuperior thoracic, which in this inftance was not wounded, generally fends off a branch, which forms an anaftomofis with the radial artery, and would, of courfe, contribute much to the carrying on of the circulation. And does not the conftant, perfect, and natural warmth of the whole limb, together with the flow of blood in the veins, for two days together, prior to the patient's death, warrant the furmife?

It may be afked, as the axillary vein was divided as high as the artery, how could the refluent blood have been taken back to the right auricle of the heart? But to this it may be anfwered, that as it would be a confiderable time, before the fmall arteries could enlarge and anaftomofe in fuch a manner, as to fupply the limb with the fame quantity of blood as it had before the accident, fo, in like manner, would the fmall veins enlarge and anaftomofe, and empty themfelves into the fubclavian. Befides, the extremities, which from their fituation are more expofed to danger, are provided with an additional fet of veins, called fubcutaneous. Thefe in the arm form the great cephalic vein, which afcends along the outer edge of the biceps, near the furface of the body, between the deltoid and pectoral mufcles, entirely out of the courfe of the wound in this cafe, and empties itfelf into the fubclavian, near the middle of the clavicle; by thefe means, the circulation in the limb is preferved, when the deep-feated veins are either obftructed from the contraction of the furrounding mufcles, or when they are divided by accident.

*Manchefter, April* 2, 1783.

